# An R2R3-MYB Transcriptional Factor *LuMYB314* Associated with the Loss of Petal Pigmentation in Flax (*Linum usitatissimum* L.)

**DOI:** 10.3390/genes15040511

**Published:** 2024-04-18

**Authors:** Dongliang Guo, Haixia Jiang, Liqiong Xie

**Affiliations:** 1 Xinjiang Key Laboratory of Biological Resources and Genetic Engineering, College of Life Science and Technology, Xinjiang University, Urumqi 830017, China; gdljhx@xju.edu.cn; 2 Key Laboratory of Plant Stress Biology in Arid Land, College of Life Science, Xinjiang Normal University, Urumqi 830017, China; jhx520gdl@163.com

**Keywords:** flax (*Linum usitatissimum* L.), petal color, genome-wide association study (GWAS), R2R3-MYB transcription factor, anthocyanin

## Abstract

The loss of anthocyanin pigments is one of the most common evolutionary transitions in petal color, yet the genetic basis for these changes in flax remains largely unknown. In this study, we used crossing studies, a bulk segregant analysis, genome-wide association studies, a phylogenetic analysis, and transgenic testing to identify genes responsible for the transition from blue to white petals in flax. This study found no correspondence between the petal color and seed color, refuting the conclusion that a locus controlling the seed coat color is associated with the petal color, as reported in previous studies. The locus controlling the petal color was mapped using a BSA-seq analysis based on the F_2_ population. However, no significantly associated genomic regions were detected. Our genome-wide association study identified a highly significant QTL (*BP4.1*) on chromosome 4 associated with flax petal color in the natural population. The combination of a local Manhattan plot and an LD heat map identified *LuMYB314*, an R2R3-MYB transcription factor, as a potential gene responsible for the natural variations in petal color in flax. The overexpression of *LuMYB314* in both *Arabidopsis thaliana* and *Nicotiana tabacum* resulted in anthocyanin deposition, indicating that *LuMYB314* is a credible candidate gene for controlling the petal color in flax. Additionally, our study highlights the limitations of the BSA-seq method in low-linkage genomic regions, while also demonstrating the powerful detection capabilities of GWAS based on high-density genomic variation mapping. This study enhances our genetic insight into petal color variations and has potential breeding value for engineering *LuMYB314* to develop colored petals, bast fibers, and seeds for multifunctional use in flax.

## 1. Introduction

Flowers represent the primary organs for plant reproduction and comprise sepals, stamens, pistils, and petals. The petal color is an important agronomic trait that plays a key role in the plant’s ecological adaptation and evolution [1,2]. The diversity of petal colors is the result of billions of years of long-term selection by pollinators. Pigmentation patterns of petals enhance insect pollination, augment the efficiency of plant pollination, and boost yields in cross-pollinated crops [3]. The petals can also have an important ornamental or edible value. Many closely related plant species exhibit varying petal hues and designs. In certain instances, alterations in the petal color or pattern may cause changes in the pollinators, resulting in reproductive isolation and species differentiation [4,5,6]. The petal color is determined by pigments found in the petal cells. In flowering plants, the predominant pigments are flavonoids, specifically anthocyanins. In addition to serving as a visual signal, anthocyanin loss is an important feature of the “domestication syndrome”, as exemplified by the regulation of convergent anthocyanin loss in cereals by a *c1/pl1* gene located within a chromosomal syntenic block [7]. Furthermore, anthocyanin pigments are essential for adaptations to diverse environmental stresses, including light, cold, drought, moisture, and microbes [8,9,10]. Plants accumulate anthocyanins in large quantities under stress [11,12]. Anthocyanins offer photoprotection to photosynthetic cells and developing organs in response to light stress [13]. Furthermore, it has been acknowledged that seeds with high levels of anthocyanin can provide health benefits and are considered healthy food items [14].

The biosynthesis of anthocyanin is one of the most clearly studied plant metabolism pathways. Red, purple, and blue anthocyanins are the products of a series of enzymatic steps that are highly conserved in plants, and they constitute the most common pigments for flower and fruit coloration [15,16]. The anthocyanin biosynthetic pathway comprises core enzymes such as chalcone synthase (CHS), chalcone isomerase (CHI), flavanone 3-hydroxylase (F3H), flavonoid 3′-hydroxylase (F3′H), flavonoid hydroxylase (F3′5′H), dihydroflavonol 4-reductase (DFR), anthocyanidin synthase (ANS), and UDP-3-O-glucosyltransferases (UF3GT). Flavonols and anthocyanin glycosides are transported and stored in vacuoles by both glutathione-s-transferase (GST) and via multidrug and toxic compound extrusion (MATE), showing a wide range of colors [17,18,19,20]. In the early steps of the anthocyanin synthesis pathway, the structural genes are regulated by the MBW ternary complex transcription factor, which consists of R2R3-MYB, basic helix–loop–helix (bHLH) transcription factors, and WD repeat protein (WDR). The MBW complex is a significant regulator involved in activating the core enzymes or genes in the flavonoid and anthocyanin pathways [21,22,23].

The MYB transcription factors (TFs) are crucial in the anthocyanin biosynthesis pathway in plants. According to the number of (1R, 2R, 3R, 4R) repeats, MYB-TFs are classified into four groups—1R-MYB, R2R3-MYB, 3R-MYB, and 4R-MYB [20,24]. The various types of MYB-TFs have different roles in regulating the anthocyanin biosynthesis pathway, with some MYB-TFs acting as activators and some as repressors [2,25,26]. The MYB transcription factors that promote anthocyanin synthesis are mainly R2R3-MYBs, while those that inhibit anthocyanin synthesis include some R2R3-MYBs and R3-MYBs [25,27]. In flowering plants, the R2R3-MYB transcription factors that activate anthocyanin biosynthesis mostly belong to subgroup 6 [27]. Their mode of action is primarily through direct binding to the promoters of anthocyanin structural genes to enhance transcription or through the co-regulation of the expression of structural genes by binding to other transcription factors, typically in the form of the MBW ternary complex [28,29]. The R2R3-MYB transcription factors that inhibit anthocyanin biosynthesis mostly belong to subgroup 4 [27,30,31]. The R3-MYB suppressor is derived from subgroup 4 of R2R3-MYB [27]. The negative effect of MYB-TFs on anthocyanin biosynthesis may primarily depend on the C-terminal motif of MYB-TFs [25]. R2R3-MYB and R3-MYB repressors inhibit anthocyanin synthesis by direct binding to the promoters of anthocyanin structural genes to repress gene expression or by inhibiting the function of the MBW complex [2,30,32,33,34].

The genus *Linum* comprises approximately 200 species [35], boasting abundant variations in petal color, such as red, yellow, blue, purple, pink, and white colors [36]. Cultivated flax (*Linum usitatissimum* L.) is one of the founding agricultural crops, which was domesticated from the wild progenitor pale flax (*Linum bienne* Mill.) approximately 30,000 years ago. Flax is an important economic crop due to the superior quality of its bast fiber and the high concentration of omega-3 α-linolenic acid (approximately 55%) in its seeds [37,38]. Previous research has classified 11 *Linum* species into yellow-flowered and blue-flowered clades, and wild flax *L. bienne* was grouped in the blue-flowered clade with blue petals [36]. The ancestral state has undergone numerous transformations, resulting in a range of petal colors including blue, purple, white, and pink in cultivated flax. Approximately 90% of cultivated flax retains a blue petal color similar to that of its wild progenitor pale flax, with white petals making up the majority of the remaining 10% or so. Regarding the genetic variation in the transition from blue to white petals, previous studies identified a locus *D* related to flax seed color via QTL mapping, which is believed to be associated with petal color variations [39]. We observed no correlation between petal color and seed coat color variations in flax, indicating their independence. Given the lack of an accurate resolution of the genetic loci of petal color variations in flax during domestication, we undertook a study to investigate the genetic basis of the transition from blue to white petals in cultivated flax.

In this study, genetic loci responding to flax petal color variations were identified using the BSA-seq and GWAS methods. A genetic analysis indicated that the petal color was controlled by a single dominant locus. The study detected a significant QTL (*BP4.1*) on chromosome 4 and identified an R2R3-MYB transcription factor (*LuMYB314*) as a candidate gene for petal coloration in flax. The overexpression of *LuMYB314* in both *Arabidopsis thaliana* and *Nicotiana tabacum* resulted in anthocyanin deposition phenotypes. The results of this study not only have important theoretical value for the regulatory mechanism of petal coloration but also have potential breeding value for the production of anthocyanin-rich seeds or colored fibers via genetic engineering of the anthocyanin biosynthetic pathway.

## 2. Materials and Methods

### 2.1. Plant Materials and Phenotyping for Petal Color

Two flax cultivars, “BETA 201” and “Tammes Type 12”, were used as parents to develop segregating populations for petal color. Tammes Type 12 is a white petal cultivar from the Netherlands, and the germplasm was obtained from the Plant Gene Resource Center in Canada (PGRC). The BETA 201 cultivar is a blue petal cultivar from Hungary, and the germplasm was obtained from the United States National Plant Germplasm System (U.S.NPGS). A cross was made between BETA 201 and Tammes Type 12 to create F_1_, and several F_1_ samples were individually self-pollinated to generate the F_2_ segregating populations. In addition, a natural population containing 24 white petal varieties and 165 blue petal varieties was used for a genome-wide association study. The F_2_ populations and associated mapping populations were planted in 2019 in an experimental field in Urumqi, Xinjiang (43°55′ N, 87°42′ E; altitude 688.0 m), and field management followed conventional cultivation practices. Flowers from each individual in the F_2_ generation and the associated mapping population were photographed during flowering and individuals were visually classified into two phenotypic classes (blue and white).

### 2.2. Construction of Segregating Pools and Sequencing

To construct two progeny pools, 20 blue petal and 20 white petal plants were selected from the F_2_ population, while two parental pools were constructed by sampling the parents. Genomic DNA was extracted individually for each sample in the progeny pool using a minor modified cetyltrimethylammonium bromide (CTAB) method [40]. The isolated DNA was quantified using a NanoDrop 2000 spectrophotometer (Thermo Scientific, Fremont, CA, USA). Two progeny DNA pools were constructed by mixing an equal amount of DNA from 20 blue petal and 20 white petal F_2_ plants, respectively. Then, the DNA was randomly fragmented into 350 bp fragments using S2/E210 Ultrasonicator (Covaris, Woburn, MA, USA). The fragments underwent end repair, the addition of A at the 3′ end, the addition of sequencing junctions, purification, and PCR amplification to construct sequencing libraries. The libraries were then qualified for sequencing using the Illumina sequencing platform (Illumina, Inc., San Diego, CA, USA). The raw sequencing data were filtered using an inhouse Perl script available with Biomarker Technologies Co. Ltd. (Beijing, China). The biparental and progeny pool high-quality sequencing data were aligned to the flax reference genome using BWA software (ver. 0.7.12) [41,42]. The variant detection methods, including SNP and small insertion–deletion (indel), were performed using GATK (ver. 3.3-0) [43]. The variant locus among the samples was summarized for the bulk segregant analysis.

### 2.3. Bulk Segregant Analysis

Both SNP index and indel index methods were used to determine the genomic regions associated with petal coloration in flax. The SNP index represents the proportion of reads containing the SNP that differ from the reference genome sequence, and the ΔSNP index was calculated as (SNP index of white petal pool)—(SNP index of blue petal pool). The indel and Δindel index values were computed using the exact same method as the SNP index. The stronger the association between the SNP or indel and the trait variation, the closer the ΔSNP or Δindel index is to 1. To eliminate false-positive loci, we calculated the average SNP index and indel index of the genome using a 4 Mb sliding window with a 10 kb step. We plotted the SNP index and indel index of the two pools, alongside the relevant ΔSNP index and Δindel index. The threshold value used to map the petal color controlled by a dominant locus was expected to be 0.667 for an F_2_ population [44,45,46].

### 2.4. Genome-Wide Association Study

We performed a genome-wide association study of blue and white variants of petal color. The petal coloration showed exhibited a consistent phenotype across the different environments, so we did not consider environmental effects in the genome-wide association study for this trait. A total of 654,174 single-nucleotide polymorphisms (SNPs; minor allele frequency ≥ 0.05) were used in the GWAS for petal coloration in the population, as described previously [47]. The genome-wide association study (GWAS) was performed using a compressed mixed linear model (MLM) with TASSEL 5.0 software [48]. The population structure (Q) and kinship matrix (K) were generated using the Admixture 1.23 and TASSEL 5.0 software programs, respectively. A stringent criterion was selected to screen the SNPs with a significant association with petal color. The threshold was defined as *p* ≤ 1.53 × 10^−8^ (0.01/*n*, where *n* is the total number of markers used) [49]. We used the R package “LDheatmap” to construct a linkage disequilibrium (LD) heatmap surrounding the candidate region on Chr4 in this GWAS [50]. The physical locations of the SNPs were identified and annotated based on flax genome sequence version ASM22429v2 (https://www.ncbi.nlm.nih.gov/datasets/genome/GCA_000224295.2/, accessed on 15 April 2024) [41].

### 2.5. Phylogenetic Analysis

Bioinformatic and phylogenetic analyses were used to infer the domain and functional conservatism of *LuMYB314*, a candidate gene identified by our pipeline as strongly associated with petal color. Compare the amino acid sequences in LuMYB314 and its homologous proteins in plant species ranging from Rhodophyta to angiosperms, including *Porphyra umbilicalis*, *Chlamydomonas reinhardtii*, *Sphagnum magellanicum*, *Selaginella moellendorffii*, *Amborella trichopoda*, *Oryza sativa*, and *Arabidopsis*. The protein sequences were obtained through BLAST searches using the Phytozome database (https://phytozome-next.jgi.doe.gov/, accessed on 15 April 2024). An amino acid alignment of the sequences was conducted using ClustalW with default parameter settings in MEGA version 5 [51]. A phylogenetic and protein similarity analysis of LuMYB314 orthologs in the plants was performed. Full-length sequences of LuMYB314 orthologs from various plants were collected using BLAST searches in the National Center for Biotechnology Information (BLAST: https://blast.ncbi.nlm.nih.gov/Blast.cgi, accessed on 15 April 2024). A phylogenetic tree was constructed using the maximum likelihood (ML) algorithm with 1000 bootstrap replicates in MEGA version 5 [51].

### 2.6. Gene Cloning and Vector Construction

The Tammes Type 12 samples were sown in nutrient soil. After 2–3 weeks of growth, the leaves were sampled and the total RNA was extracted using the RNAprep Pure Plant Kit (TIANGEN). The mRNA was then reversed to cDNA using the Reverse Transcription Kit (5× All-In-One RT MasterMix) after passing the quality control test. Primers were designed specifically for the full-length coding sequence (CDS) of *LuMYB314* based on the flax reference genome sequence in the Phytozome database (http://www.phytozome.net, accessed on 15 April 2024) (Table S1). The CDS of *LuMYB314* was amplified from flax cDNA using a high-fidelity enzyme (TransTaq^®^ DNA Polymerase High Fidelity (HiFi)). Afterward, a PCR product was ligated into the *pEASY*^®^-T5 Zero Vector and transformed into *Escherichia coli* DH5ɑ, and sections of randomly selected individual colonies were sequenced at Sangon Biotech (Shanghai, China) Co., Ltd. After sequencing and verifying the sequence of *LuMYB314*, the T5 vector and pCAMBIA2300 vector were cleaved using the restriction endonucleases *Kpn*I and *Xba*I, respectively. Subsequently, *LuMYB314* was ligated into the pCAMBIA2300 vector using T4 ligase, and the overexpression vector was validated using PCR, digestion, and sequencing. The overexpression vector was transformed into the *Agrobacterium rhizogenes* strain GV3101.

### 2.7. Transgenic Analysis

*Arabidopsis* Columbia-0 (Col-0) and *Nicotiana* NC89 were chosen as the recipient materials for the genetic transformation. The overexpression vector was transformed into *Nicotiana* NC89 using *Agrobacterium rhizogenes* GV3101, following a modified genetic transformation method [52]. Positive *Nicotiana* transgenic lines were confirmed using a polymerase chain reaction (PCR) analysis. The overexpression vector was transformed into *Arabidopsis* Col-0 by *Agrobacterium*-mediated floral dipping at the flowering stage, and the positive transgenic plants were screened with MS medium containing 50 mg·L^−1^ of Kanamycin and tested via PCR. The anthocyanins were extracted with 1% (*v*/*v*) HCl/methanol and the total anthocyanin content was measured as previously described [53].

## 3. Results

### 3.1. Patterns of Variation in Petal Color

**Figure 1:**
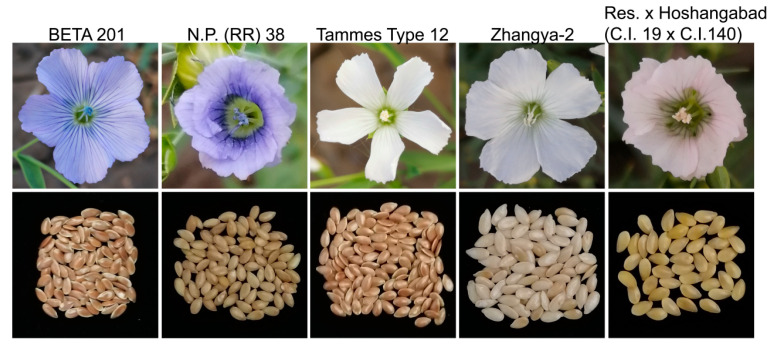
Variations in petal color and seed coat color in several representative varieties in flax. The figure displays the variety names at the top, with each variety’s flowers (**top**) and seeds (**bottom**) showing a one-to-one correspondence.

Figure 1 presents the variations in petal and seed coat color of several representative flax varieties. It is obvious that some varieties with blue petals have brown seeds, while others are yellow. Similarly, some white petal varieties have brown seeds, while others have light yellow seeds. These results indicate that there is no direct correlation between the petal color and seed coat color, indicating that the genetic loci regulating the flax petal color and seed coat color are separate. A hybrid of Tammes Type 12 with white petals and BETA 201 with blue petals resulted in an F_1_ generation with blue petals. Upon self-crossing the F_1_ generation, a segregation of the petal colors was observed in the F_2_ population. An analysis of single-plant self-crosses in the F_2_ generation showed that the segregation ratio of blue petal (261) and white petal (76) samples was in line with the expected 3:1 ratio (chi-square test, *p* = 0.30). These results suggest that a single dominant locus controls the variation in blue and white petals in flax.

### 3.2. Whole-Genome Resequencing and Bulk Segregant Analysis

**Figure 2:**
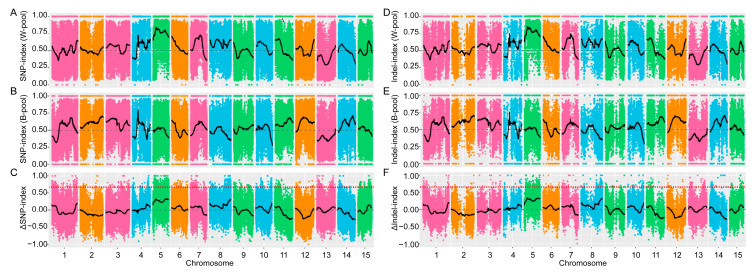
Bulk segregation analyses of the petal coloration in the F_2_ segregating population. The horizontal axis indicates the physical positions of 15 chromosomes in flax and the vertical axis indicates the SNP/indel index or ΔSNP/Δindel index. Each SNP/indel site is plotted as a colored dot, and the black lines represent the fitting results. (**A**,**B**) SNP index values of the white petal pool (**A**) and blue petal pool (**B**). (**C**) The ΔSNP index. (**D**,**E**) Indel index values of the white petal pool (**D**) and blue petal pool (**E**). (**F**) The ΔIndel index. The red dotted lines indicate the association threshold (0.667).

The Illumina high-throughput sequencing produced 35,419,168 and 35,232,344 raw reads from Tammes Type 12 and BETA 201, respectively. After eliminating adapters, low-quality reads, and N ratios above 10%, the numbers of clean reads were reduced to 35,294,855 and 35,132,913, respectively. Similarly, the sequencing produced 31,522,547 and 41,103,457 raw reads from the white and blue petal pools, respectively. These were then filtered resulting in 31,444,722 and 41,002,462 clean reads. The filtered sequencing data yielded 42.79 Gb of clean bases, with a Q30 of 80%, an average sequencing depth of 33.84× per sample, an average sample-to-reference genome match rate of 85.03%, an average coverage depth of 17.25×, and a genome coverage rate of 98.87%. A combined total of 667,141 SNPs and 122,925 indels were identified between the parental plants, while the two mixed pools originating from the F_2_ generation yielded a combined total of 199,922 SNPs and 43,708 small indels. Subsequently, the SNP index and the indel index were used to identify the genomic regions associated with the petal color variants. Figure 2 presents the distribution characteristics of the SNP and indel index values, as well as the ΔSNP and Δindel index values. The results showed that the ΔSNP and Δindel index values were well below the thresholds (Figure 2C,F), and no genomic regions associated with petal coloration were obtained using the BSA-seq method.

### 3.3. Identification of a Major QTL for Petal Coloration via a GWAS

**Figure 3:**
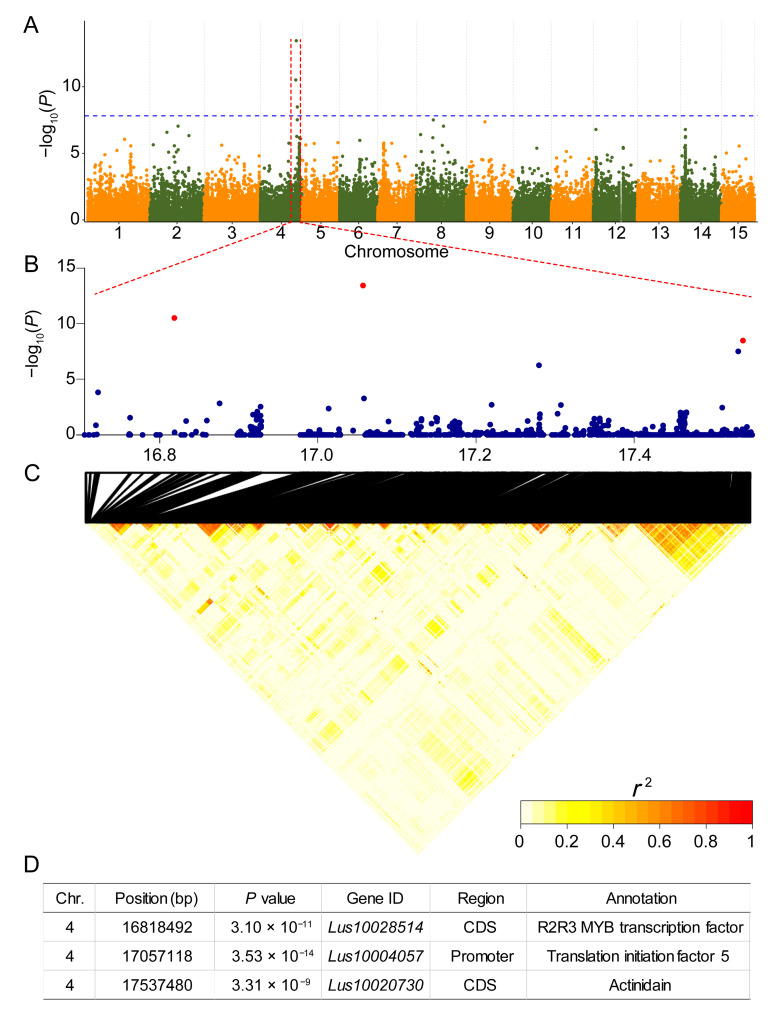
Genome-wide association study of petal coloration in flax. (**A**) Manhattan plots illustrating a significantly associated signal for petal coloration using the compressed mixed linear model (MLM). The blue horizontal dash-dot line indicates the genome-wide significant threshold (1.53 × 10^−8^). The vertical axis indicated the −log_10_(*P*) value. (**B**) Local Manhattan plot of petal coloration surrounding the associated signal peaks on chromosome 4. The three red labelled points indicate SNPs above the threshold. (**C**) LD heat map drawn around the peak on chromosome 4. Representations of the pairwise *r*^2^ values among all SNPs in the genomic region corresponding to (**B**). (**D**) Detailed annotation information for three significant SNPs.

We were unable to detect the locus associated with flax petal color variations based on the BSA-seq of the F_2_ linkage population. Therefore, we attempted to identify causal variations in flax petal color using an association population. We performed a genome-wide association study (GWAS) using 165 blue petal and 24 white petal varieties, and detected the only strong association signal on chromosome 4 (Chr.4:17057118, *p* < 3.53 × 10^−14^), naming this locus *BP4.1* (*Blue Petal 4.1*) (Figure 3A). Subsequently, we plotted local Manhattan plots and LD heat maps for this region (Figure 3B,C), which showed an extremely weak linkage, with only three SNPs above the threshold, which were far apart (Figure 3B). The LD values were calculated for the three significant SNPs, and the results revealed their strong linkage (*r*^2^ of 0.68, 0.35 and 0.42, respectively), suggesting that the three SNPs showed long-distance linkage (Figure 3C). Subsequently, each of the 3 significant SNPs was annotated (Figure 3D). The SNP on Chr.4:16818492 is located in the exon region of *Lus10028514*, which encodes an MYB transcription factor. The SNP Chr.4:17057118 is located within the promoter region of *Lus10004057*, which encodes a translation initiation factor 5. The SNP Chr.4:17537480 is located within the exon region of *Lus10020730*, the gene responsible for encoding senescence-associated gene 12 (SAG12). *Lus10028514* (*LuMYB314*) belongs to the G16 subgroup of R2R3-LuMYBs. G16 corresponds to subgroup 6 in *Arabidopsis*, and the members of subgroup 6 are closely associated with anthocyanin biosynthesis. Therefore, it can be inferred that *LuMYB314* is a candidate gene of the *BP4.1* locus.

### 3.4. The Novel LuMYB314 Belongs to a Clade of R2R3 MYB Transcription Factors

**Figure 4:**
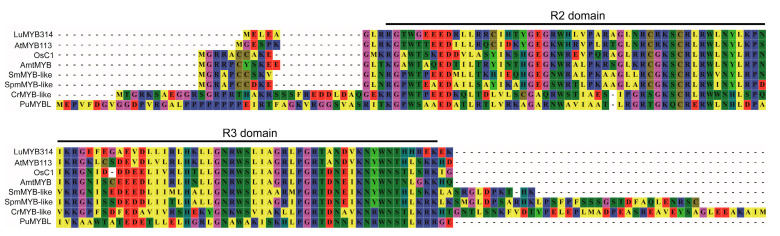
Multiple alignments of the amino acid sequences in the R2R3 region of LuMYB314 (Lus10028514) and its homologous proteins in plant species ranging from Rhodophyta to angiosperms. Taxon names are abbreviated as follows: Lu, *Linum usitatissimum*; At, *Arabidopsis thaliana*; Os, *Oryza sativa*; Amt, *Amborella trichopoda*; Sm, *Selaginella moellendorffii*; Spm, *Sphagnum magellanicum*; Cr, *Chlamydomonas reinhardtii*; Pu, *Porphyra umbilicalis*.

The full-length coding sequence (CDS) of *LuMYB314* was amplified from Tammes Type 12, a white petal flax variety. The resulting CDS sequence was 819 bp and was aligned to the flax reference genome CDC Bethune, a blue-flowering variety (Figure S1). No variants were detected, indicating that the causative variants are not in the CDS region. *LuMYB314* encodes an MYB transcription factor that consists of 272 amino acids (Figure S2). Multiple alignments of the amino acid sequences of LuMYB314 and its homologous proteins in plant species ranging from Rhodophyta to angiosperms showed that LuMYB314 contains the typical R2 and R3 MYB DNA-binding domains at the N-terminus (Figure 4). These domains are conserved by the R2R3-MYB transcription factor family during plant evolution. *LuMYB314* is identified as a candidate gene in this study that belongs to the R2R3-MYB transcription factor family.

**Figure 5:**
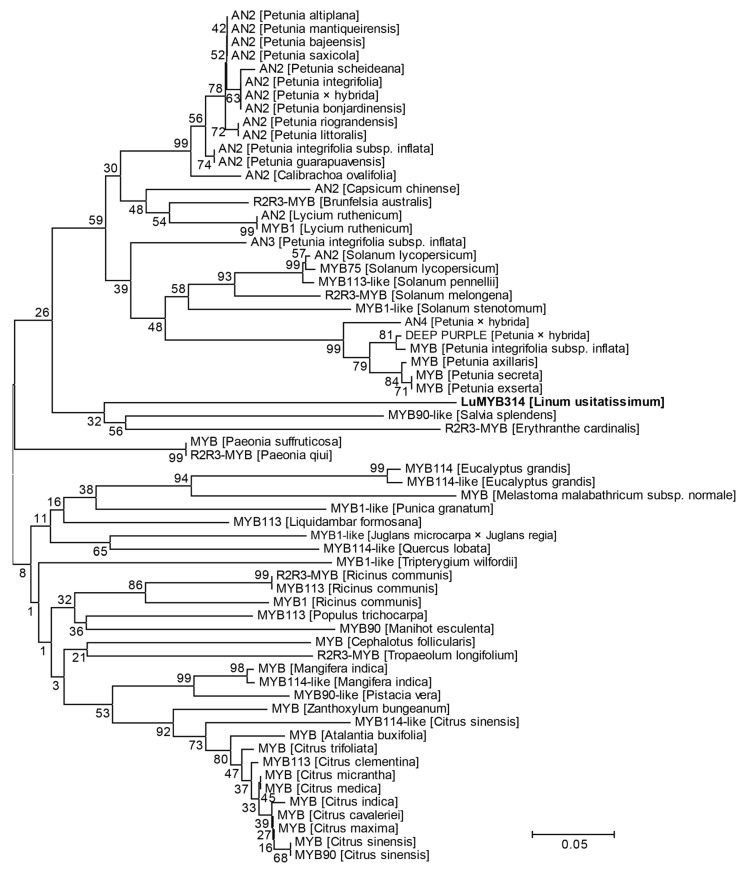
Phylogenetic tree of LuMYB314 homologs in plants. Amino acid sequences of LuMYB314 homologs from various plants were collected using BLAST in NCBI. This phylogenetic tree was constructed using the maximum likelihood method. A bootstrap analysis was performed with 1000 replications and the values are expressed as percentages. The proteins marked in bold on the evolutionary tree correspond to LuMYB314 identified in this study. Scale bar indicates the distances in substitutions per amino acid.

A phylogenetic analysis revealed that the LuMYB314 protein was closely related to the MYB90-like transcription factor of *Salvia splendens* and the R2R3-MYB transcription factor of *Erythranthe cardinalis* (Figure 5). The MYB90-like transcription factor in *Salvia splendens* is a predicted gene in the reference genome that has not been functionally studied. The R2R3-MYB transcription factor in *Erythranthe cardinalis* regulates the production of anthocyanin in petals [54]. Furthermore, the branch where the LuMYB314 protein is located is predominantly occupied by the anthocyanin 2 (AN2) protein (Figure 5). AN2 is a well-defined MYB transcription factor that plays a significant role in the variations in flower color across multiple species. The results suggest that *LuMYB314* may be a plausible candidate gene for controlling petal coloration in flax. However, LuMYB314 is phylogenetically distant from the AN2 protein, so further verification is necessary to determine whether this gene is associated with anthocyanin deposition.

### 3.5. LuMYB314 Associated with Anthocyanin Deposition

**Figure 6:**
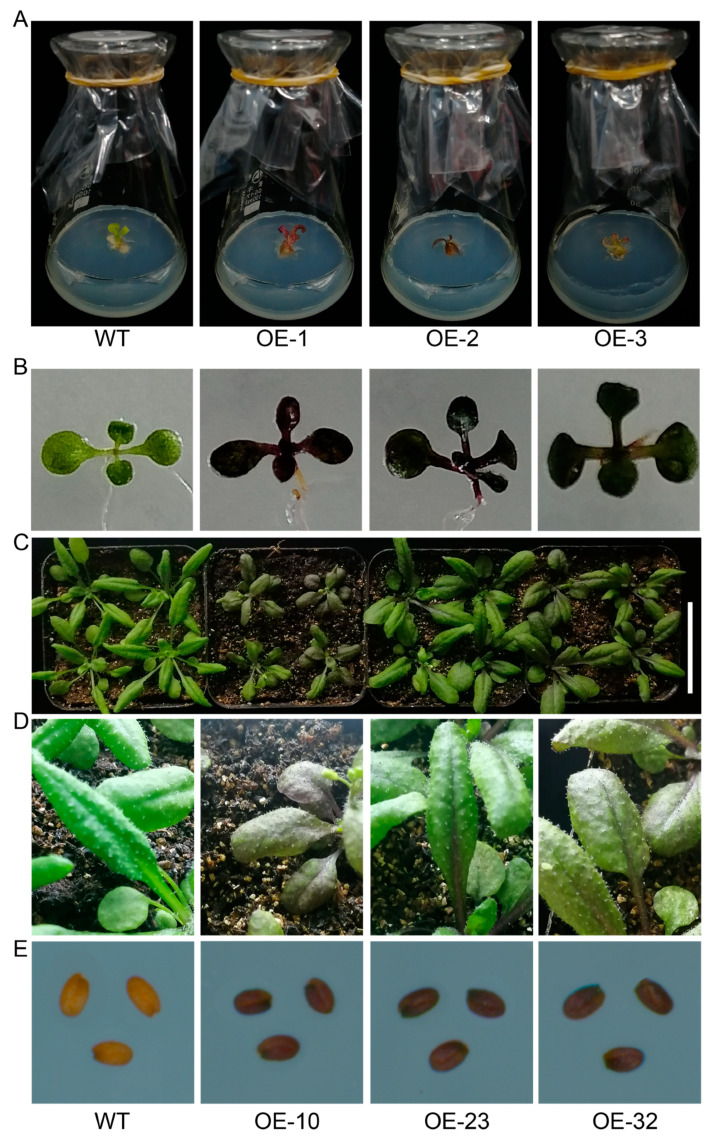
Overexpression of *LuMYB314* increased anthocyanin accumulation in *Nicotiana tabacum* and *Arabidopsis*. (**A**) Anthocyanin deposition phenotype of wild-type *Nicotiana* NC89 and three independent *35S:LuMYB314_NC89_* transgenic *Nicotiana* tissue culture seedlings. (**B**–**E**) Anthocyanin deposition phenotype of wild-type (Col-0) and three independent *35S:LuMYB314_Col-0_* transgenic *Arabidopsis* samples. (**B**) Seedlings. (**C**) Rosette leaf (scale bar, 5 cm). (**D**) A close-up of the rosette leaf. (**E**) Mature seeds.

To further verify the function of *LuMYB314*, we overexpressed the *LuMYB314* in *Nicotiana* and *Arabidopsis*. Figure 6 shows that the overexpression of *LuMYB314* led to an increase in anthocyanin accumulation in both *Nicotiana* and *Arabidopsis*. *Nicotiana* seedlings overexpressing *LuMYB314* showed significant anthocyanin deposition compared with the wild type (Figure 6A). Subsequently, we obtained a transgenic *Arabidopsis* line, the *35S:LuMYB314* plant, which produced enough anthocyanins to make the color visible (Figure 6B–E), and their anthocyanin content was significantly increased (Figure S3). Anthocyanin accumulation was observed in all tissues of *Arabidopsis* throughout its life cycle. A strong anthocyanin deposition phenotype was observed in the seedlings, leaves, leaf veins, and seeds of the *35S:LuMYB314* plants (Figure 6B–E). In summary, these results suggest that *LuMYB314* is involved in anthocyanin synthesis, and the regulatory pathway of *LuMYB314* may be relatively conserved in plants. Therefore, it can be inferred that *LuMYB314* is associated with natural variations in petal color in flax.

## 4. Discussion

### 4.1. Natural Variations in Flax Petal Color Were Not Related to Variations in Seed Coat Color

This study investigated the correlation between the natural variations in petal color and seed coat color in flax. Through an investigation and analysis of numerous flax varieties, it was discovered that there is no clear correlation between variations in petal color and seed coat color. In other words, there was no one-to-one correspondence between the petal color and seed coat color. In a previous study, a hybrid population was conducted between a brown-grained, blue-flowered variety and a yellow-grained, white-flowered variety to map a seed coat color-related locus *D*, which is thought to be associated with petal color variations in flax [39]. The locus *D* determines both the seed coat and petal colors, with brown seeds corresponding to blue petals and yellow seeds corresponding to white petals [39]. The study suggested that the genetic loci regulating flax petal and seed coat color variations are separate. The previously identified *D* locus may only be associated with seed coat color variations, as the *D* locus was mapped based on seed coat color variations in the recombinant inbred population [39]. This study shows that the blue–white variation in flax petals is controlled by a single dominant gene, *LuMYB314*, located on chromosome 4. In plants, the deposition of anthocyanin in specific or multiple tissues is related to the functional specificity and spatiotemporal expression patterns of anthocyanin-related genes. For instance, the natural variation in an R2R3-MYB gene (*c1/pl1*) results in the convergent decolorization of various organs in cereal crops, including the roots, stems, leaves, panicles, and seeds [7]. In the same natural soybean population, the loci that regulate variations in flower color are distinct from those that regulate variations in seed coat color, and they are located on different chromosomes [49]. Based on the expression profile information generated from the RNA-seq data, *LuMYB314* is specifically expressed only in flowers and buds [4]. Thus, it is hypothesized that the natural variation in *LuMYB314* may solely be associated with petal coloration.

Interestingly, a study of flax R2R3-LuMYB family members, based on qRT-PCR experiments, found that the expression patterns of all seven members of the S5 and S6 subgroups associated with anthocyanin synthesis were consistent with a deepening of the flax flower color, with the highest level of expression being found in *LuMYB216* [55]. The gene editing of *LuMYB216* in flax significantly reduced the accumulation of anthocyanins in the petals and seed coat [55]. Based on the expression profile information generated from the RNA-seq data, it is noted that *LuMYB216* is expressed specifically in seeds [55]. The petal color of *LuMYB216* gene-edited plants changed from blue to a lighter shade, although they did not produce white petals [55]. Thus, we hypothesize that *LuMYB216*, while involved in flax anthocyanin synthesis, is not the allele that determines the natural variation in petal color from blue to white. Overall, this study demonstrates that the genetic loci for petal color and seed coat color in flax are distinct and that *LuMYB314* is associated with a loss of petal pigmentation in flax.

### 4.2. In Some Weakly Linked Genomic Regions, GWAS Is More Powerful Than BSA-Seq for QTL Detection

Currently, linkage-based QTL mapping and linkage disequilibrium (LD)-based association mapping are the two most effective methods for functional gene mapping. Compared to the linkage mapping populations, the association mapping population typically employs a diverse natural population that has undergone a long process of domestication and improvement, resulting in a reduction in the extent of LD through continuous recombination events, thereby allowing more precise mapping of functional genes [49,56]. The extent of the LD varies significantly across genomic regions [49,57], which affects the size of the detected QTL intervals. This allows for mapping at both the single-gene level or QTL intervals up to the Mb level within the same genome [58]. In this study, we constructed an F_2_ population and attempted to map genes related to flax petal coloration using BSA-seq. BSA-seq has strong detection ability in mapping genes related to traits controlled by a single gene [56,59], and surprisingly we did not detect any significantly associated locus (Figure 2). A genome-wide association study approach was subsequently chosen to identify the genetic locus associated with petal color variations in flax. A significant association locus was mapped on chromosome 4 (*BP4.1*). Notably, only three SNPs were found to be above the threshold at the *BP4.1* locus. The LD heat map around the *BP4.1* locus showed that the linkage in this region was extremely weak. This may be the main reason why the genetic locus was not detected in this genomic region using the BSA-seq method. The BSA method is based on the generation of a regular biased distribution of SNP/indel index values within a genomic region to detect QTLs. Therefore, this method heavily depends on SNP/indel markers that have a higher extent of LD within the candidate genomic region. For instance, a genome-wide association study approach detected the rind-color-related locus *Dgo* on chromosome 4 in watermelon, with only two SNPs surpassing the threshold [60]. In contrast, the *Dgo* locus was difficult to detect using the BSA-seq method when calculating the ΔSNP index [61]. These results suggest that the detection power of GWAS methods based on high-density genome variation maps is much stronger than classical BSA-seq when targeting genomic regions with extremely weak linkages. Notably, the QTL mining in regions of weaker linkage is highly dependent on high-density genomic variation datasets. A limited variation locus may result in a loss of the key locus, making it easy to miss QTLs. Of course, these weakly linkage regions are advantageous for the rapid mapping of candidate genes. The weak linkage at the *BP4.1* locus in this study enabled us to quickly target the candidate gene *LuMYB314*, and the transgene functional validation led us to conclude that *LuMYB314* is a causally variable gene for the control of petal coloration in flax.

### 4.3. The Subgroup 6 of R2R3-MYB Transcription Factors Is Conserved in Anthocyanin Biosynthesis

R2R3-MYB transcription factors are widely distributed throughout the plant kingdom, and their numbers have increased significantly during the evolution of green plants. As a result, they form one of the largest and most diverse families of transcription factor genes in land plants, especially in angiosperms [62,63,64]. The functions of R2R3-MYBs can be classified into three main processes—development and cell differentiation, metabolite biosynthesis, and stress response. To date, plant R2R3-MYBs have been predominantly reported to regulate metabolite biosynthesis, accounting for approximately 70% of its known functions [62]. The R2R3-MYB gene family has undergone expansion and functional diversification during its evolution, resulting in the formation of different subgroups. The subgroups mainly involved in the regulation of flavonoid biosynthesis are S6, S5, S4, S7, S44, and S79 [62]. Subgroup 6 is primarily responsible for anthocyanoside biosynthesis [65], as seen in *DcMYB113* [66], *GaPC* [67], and *SmMYB113* [68,69]. The candidate gene *LuMYB314*, which is associated with flax petal color variations, was mapped in this study. *LuMYB314* is a member of the R2R3-MYB transcription factor subgroup 6 [55]. The overexpression of the *LuMYB314* gene in *Nicotiana* and *Arabidopsis* resulted in transgenic plants showing an anthocyanin-depositing phenotype (Figure 6). *LuMYB314* is a homolog of *AtMYB113* in *Arabidopsis*. Homologs of *AtMYB113* are involved in regulating anthocyanin biosynthesis in various crops. For example, *GaPC*, the homolog of *AtMYB113* in cotton, is a crucial gene that regulates petal color formation in cotton [67]. The gene *DcMYB113*, which is homologous to *AtMYB113*, regulates anthocyanin biosynthesis and modifications in carrots [66]. The results suggest that the functions of subgroup 6 members involved in anthocyanin biosynthesis are relatively conserved and that *LuMYB314* is a key gene in the regulation of petal coloration in flax. Interestingly, subgroup 6 appears to have formed after the divergence of angiosperms from other vascular plants [70]. The number of R2R3-MYB genes increased significantly after angiosperms diverged from other vascular plants [63,71]. Most of the new or derived R2R3-MYB subgroups in angiosperms are metabolically related [62]. It is possible that the emergence of subgroup 6 occurred during a period of rapid expansion and functional differentiation or specialization of R2R3-MYB, and potentially at a later time. Perhaps this is why subgroup 6 members are relatively conserved in their function in anthocyanin biosynthesis in higher plants.

## 5. Conclusions

In this study, we found that a single dominant locus is responsible for the variations in blue and white petals in flax. A QTL (*BP4.1*) significantly associated with flax petal coloration was mapped on chromosome 4 via a GWAS, and an R2R3-MYB transcription factor *LuMYB314* was identified as a candidate gene for petal coloration. Both *Arabidopsis* and *Nicotiana* overexpressing *LuMYB314* exhibited a phenotype of anthocyanin deposition, suggesting that this gene is a credible candidate for petal coloration. Furthermore, our study has shown that BSA-seq methods have limited detection ability in low-linkage genomic regions. In contrast, our GWAS based on high-density genomic variation maps demonstrated powerful detection ability. Due to the limitations of the flax’s genetic transformation method, we have only been able to provide preliminary evidence for the function of *LuMYB314* in *Arabidopsis* and *Nicotiana*, and further research is needed to fully understand the function and molecular mechanism of *LuMYB314* in flax petal coloration. This study has deepened our genetic understanding of flax petal color variations and provided engineering genes to develop colored flax petals with commercial value or endogenous naked-eye reporter genes in flax.

## Data Availability

The whole-genome resequencing data that support the findings of this study are openly available and can be found in the NCBI using accession number PRJNA590636 (https://www.ncbi.nlm.nih.gov/bioproject/PRJNA590636, accessed on 15 April 2024).

## Supplementary Materials

The following supporting information can be downloaded at https://www.mdpi.com/article/10.3390/genes15040511/s1: Figure S1: Comparison of CDS sequences of *LuMYB314* between CDC Bethune (reference genome) and Tammes Type 12. Figure S2. Comparison of amino acid sequences of LuMYB314 between CDC Bethune (reference genome) and Tammes Type 12. Figure S3. Total anthocyanin contents in *Arabidopsis* seedlings of WT and three independent *35S:LuMYB314_Col-0_* transgenic lines. Table S1. The list of primers used in this study.
