# Supplementary material for: An R2R3-MYB Transcriptional Factor LuMYB314 Associated with the Loss of Petal Pigmentation in Flax (Linum usitatissimum L.)
Source: Genes (Basel). 2024 Apr 18;15(4):511. doi: 10.3390/genes15040511 (PMC11050253; doi:10.3390/genes15040511)
Supplement: Supplementary file 1 [file genes-15-00511-s001.zip › genes-2953211-supplementary figures.pdf]

Supplementary Figures:

An R2R3-MYB transcriptional factor *LuMYB314* associated with the loss of petal pigmentation in flax (*Linum usitatissimum* L.)

Dongliang Guo [1], Haixia Jiang [2] and Liqiong Xie [1*]

[1] Xinjiang Key Laboratory of Biological Resources and Genetic Engineering, College of Life Science and Technology, Xinjiang University, Urumqi 830017, China
[2] Key Laboratory of Plant Stress Biology in Arid Land, College of Life Science, Xinjiang Normal University, Urumqi 830017, China
[*] Correspondence: picea@xju.edu.cn

```
CDC Bethune      ATGGAATTAGAAGCAGGGTTACGGAGAGGAACATGGGGTGAAGAGGAGGATAGGCTGCTCCGGAGGTGCATTCACACTTA   80
Tammes Type 12   ATGGAATTAGAAGCAGGGTTACGGAGAGGAACATGGGGTGAAGAGGAGGATAGGCTGCTCCGGAGGTGCATTCACACTTA   80

CDC Bethune      TGGAGAAGGAAGATGGCATCTCGTTCCCGCCCGAGCAGGATTGAATAGATGCAGGAAAAGTTGTAGACTGAGGTGGCTGA   160
Tammes Type 12   TGGAGAAGGAAGATGGCATCTCGTTCCCGCCCGAGCAGGATTGAATAGATGCAGGAAAAGTTGTAGACTGAGGTGGCTGA   160

CDC Bethune      ATTACTTGAAGCCAAACATCAAAAGAGGAGAGTTTGAAGGAGCTGAAGTTGATCTACTCATTAGGTTGCACAAGCTTTTG   240
Tammes Type 12   ATTACTTGAAGCCAAACATCAAAAGAGGAGAGTTTGAAGGAGCTGAAGTTGATCTACTCATTAGGTTGCACAAGCTTTTG   240

CDC Bethune      GGTAACAGATGGTCGCTAATCGCCGGGCGTTTGCCGGGAAGGACGGCCAACGACGTGAAAAACTATTGGAACACCCACCA   320
Tammes Type 12   GGTAACAGATGGTCGCTAATCGCCGGGCGTTTGCCGGGAAGGACGGCCAACGACGTGAAAAACTATTGGAACACCCACCA   320

CDC Bethune      CAGGGAGAAGGAGAAGAAGAAACTAACCACCATCACGACGTCGTCCTCCATGCCTGCGGCTGCGTTGACTTTTCCATGCA   400
Tammes Type 12   CAGGGAGAAGGAGAAGAAGAAACTAACCACCATCACGACGTCGTCCTCCATGCCTGCGGCTGCGTTGACTTTTCCATGCA   400

CDC Bethune      AAAGACAGCGACATTGTACGGAACCACCGGTGGTCATCGTGGAAAAAGGAAAGATAACAAAGACCAACATCATAAGACCG   480
Tammes Type 12   AAAGACAGCGACATTGTACGGAACCACCGGTGGTCATCGTGGAAAAAGGAAAGATAACAAAGACCAACATCATAAGACCG   480

CDC Bethune      AGGCCTGTGGGCTTGCCCAAAGGATTCTGTTGGCGGGCCGAAACAAATAAGCAGGCCCAGAGCCGATGTGTTGCTGCTGG   560
Tammes Type 12   AGGCCTGTGGGCTTGCCCAAAGGATTCTGTTGGCGGGCCGAAACAAATAAGCAGGCCCAGAGCCGATGTGTTGCTGCTGG   560

CDC Bethune      TGATATTAACGGCGGCGGAAGTAGTACCGTTTGGGCACAAGAACTGAAGAGGTTTTTGGAGGATGACGTGGATAAATTCC   640
Tammes Type 12   TGATATTAACGGCGGCGGAAGTAGTACCGTTTGGGCACAAGAACTGAAGAGGTTTTTGGAGGATGACGTGGATAAATTCC   640

CDC Bethune      CAGCAGCCACCTGCAACAAAATGACAGAACATGATAATAGTAGTTGTAGTAATGATGAGATTATGAAGTTGGGTGTCGAG   720
Tammes Type 12   CAGCAGCCACCTGCAACAAAATGACAGAACATGATAATAGTAGTTGTAGTAATGATGAGATTATGAAGTTGGGTGTCGAG   720

CDC Bethune      GGTTTGACGATGTTTGACCGTCAGGAGGAAGGCAATCATCAAAGTTGTTGGGGGGATGATGATGTAAGCATTTGGGAGCT   800
Tammes Type 12   GGTTTGACGATGTTTGACCGTCAGGAGGAAGGCAATCATCAAAGTTGTTGGGGGGATGATGATGTAAGCATTTGGGAGCT   800

CDC Bethune      CCTTCAGCGATCATCTTGA   819
Tammes Type 12   CCTTCAGCGATCATCTTGA   819
```

Figure S1. Comparison of CDS sequences of *LuMYB314* between CDC Bethune (reference genome) and Tammes Type 12.

```
CDC Bethune       MELEAGLRRGTWGEEEDRLLRRCIHTYGEGRWHLVPARAGLNRCRKSCRLRWLNYLKPNIKRGEFEGAEVDLLIRLHKLL   80
Tammes Type 12    MELEAGLRRGTWGEEEDRLLRRCIHTYGEGRWHLVPARAGLNRCRKSCRLRWLNYLKPNIKRGEFEGAEVDLLIRLHKLL   80

CDC Bethune       GNRWSLIAGRLPGRTANDVKNYWNTHHREKEKKKLTTITTSSSMPAAALTFPCKRQRHCTEPPVVIVEKGKITKTNIIRP  160
Tammes Type 12    GNRWSLIAGRLPGRTANDVKNYWNTHHREKEKKKLTTITTSSSMPAAALTFPCKRQRHCTEPPVVIVEKGKITKTNIIRP  160

CDC Bethune       RPVGLPKGFCWRAETNKQAQSRCVAAGDINGGGSSTVWAQELKRFLEDDVDKFPAATCNKMTEHDNSSCSNDEIMKLGVE  240
Tammes Type 12    RPVGLPKGFCWRAETNKQAQSRCVAAGDINGGGSSTVWAQELKRFLEDDVDKFPAATCNKMTEHDNSSCSNDEIMKLGVE  240

CDC Bethune       GLTMFDRQEEGNHQSCWGDDDVSIWELLQRSS  272
Tammes Type 12    GLTMFDRQEEGNHQSCWGDDDVSIWELLQRSS  272
```

Figure S2. Comparison of amino acid sequences of LuMYB314 between CDC Bethune (reference genome) and Tammes Type 12.

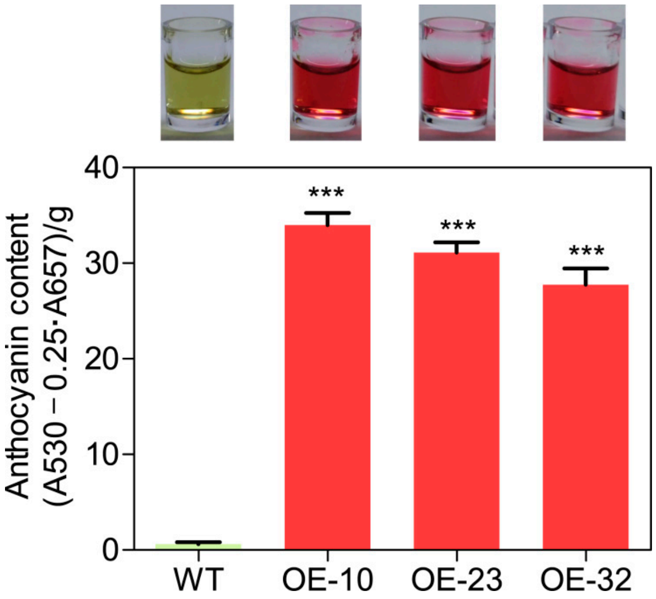

Figure S3. Total anthocyanin contents in *Arabidopsis* seedlings of WT and three independent *35S:LuMYB314*$_{Col\text{-}0}$ transgenic lines. Asterisks indicate significant differences to the wild-type Col-0 (Student's *t*-test, ***$P < 0.001$; values are means ± SD ($n = 3$)).
