# Supplementary material for: An R2R3-MYB Transcriptional Factor LuMYB314 Associated with the Loss of Petal Pigmentation in Flax (Linum usitatissimum L.)
Source: Genes (Basel). 2024 Apr 18;15(4):511. doi: 10.3390/genes15040511 (PMC11050253; doi:10.3390/genes15040511)
Supplement: Supplementary file 1 [file genes-15-00511-s001.zip › genes-2953211-Supplementary Tables.pdf]

Table S1. The list of primers used in this study.

Name	Forward primer (5′-3′)	Reverse primer (5′-3′)
LuMYB314 for overexpression vector	GGGGTACCATGGAATTAGAAGCAGGGTTACG	GCTCTAGATTAAGATGATCGCTGAAGGAGC

Enzyme cleavage sites and guard bases are marked in blue.
